# Recent Development of Nuclear Molecular Imaging in Thyroid Cancer

**DOI:** 10.1155/2018/2149532

**Published:** 2018-05-21

**Authors:** Huiting Liu, Xiaoqin Wang, Ran Yang, Wenbing Zeng, Dong Peng, Jason Li, Hu Wang

**Affiliations:** ^1^Department of Nuclear Medicine, Chongqing Three Gorges Central Hospital, Wanzhou 404000, China; ^2^Clinical Test Center, Chongqing Three Gorges Central Hospital, Wanzhou 404000, China; ^3^Department of Radiology, Chongqing Three Gorges Central Hospital, Wanzhou 404000, China; ^4^Institute for Cell Engineering, Johns Hopkins University School of Medicine, Baltimore, MD 21205, USA; ^5^Medical School, China Three Gorges University, Yichang 443002, China

## Abstract

Therapies targeting specific tumor pathways are easy to enter the clinic. To monitor molecular changes, cellular processes, and tumor microenvironment, molecular imaging is becoming the key technology for personalized medicine because of its high efficacy and low side effects. Thyroid cancer is the most common endocrine malignancy, and its theranostic radioiodine has been widely used to diagnose or treat differentiated thyroid cancer. This article summarizes recent development of molecular imaging in thyroid cancer, which may accelerate the development of personalized thyroid cancer therapy.

## 1. Introduction

Thyroid cancer is one of the most common malignant tumors in the endocrine system. Thyroid cancer is classified into differentiated thyroid cancer (DTC), medullary, and anaplastic types [1]. With the improvement of medical technology, thyroid cancer incidence is increasing all over the world [2]. Research has shown a widespread and persistent increase in thyroid. Different histological types of thyroid carcinoma have different biological behaviors and prognoses. DTC are mild tumors with good prognosis and long-term survival, while the other major thyroid cancers tend to be more aggressive and deadly. However, there are exceptions, such as Hürthle cell carcinoma in DTC with a lower survival rate, whereas medullary types have high survival rate. It is necessary to recognize the degree of malignancy and determine the appropriate therapy and prevention from disease recurrence.

Molecular imaging plays an important role in diagnosing and managing thyroid cancer, because it allows visual representation, characterization, and quantification of the biological characteristics of cells and tissues in the patients [3] and also helps ensure that patients get the optimal medical therapy for their disease or personalized treatment. Of all the molecular imaging methods, molecular nuclear medicine has made advances rapidly in both diagnosis and treatment of thyroid cancer. Molecular nuclear imaging especially can semiquantitatively or quantitatively demonstrate the alterations in specific molecules of thyroid cancer on cellular and molecular level. Moreover, multimodal nuclear imaging is essential to design the lesion-based multimodal treatment strategy for patients with multiple heterogeneous metastatic lesions [4]. Here we will summarize the application of nuclear imaging in thyroid cancer and discuss the latest progresses within this context.

## 2. Nuclear Molecular Imaging in DTC

Slow growth potential, strong survival, and good clinical outcomes characterize DTC, because of its benign biological behaviors and good responses to proper medical therapies. Even with metastasis, as long as adequately treated with effective therapeutic approaches (total and subtotal thyroidectomy, radioactive iodine and thyroid hormone suppression), these tumors' 10-year survival rates are above 90% [5, 6]. Fortunately, DTC accounts for the vast majority of thyroid cancers (about 80% to 90%), and DTC includes papillary (about70%), follicular carcinoma (about 20%), and Hürthle cell (about 10%) [1]. Nevertheless, dedifferentiation, local-regional recurrence, or distant metastasis can be observed in some patients and lead to a poor prognosis. Various advanced imaging technologies can detect these lesions early and help in making clinical decisions.

### 2.1. Radioiodine Molecular Imaging in DTC and Metastasis

Theragnostic is an invaluable tool in personalized medicine using diagnostic testing to detect molecular targets for particular therapeutic modalities for different patients, even for individual lesions in a patient [4]. Radioiodine, the first theragnostic agent, was used on DTC and metastases [7]. Radionuclide scintigraphy and therapy with 123I/131I are used in the treatment and follow-up of patients with DTC [8]. Radionuclide therapy has the advantage of delivering a highly concentrated dose to the targeted tumor while not intruding the surrounding normal tissues [9]. The uptake mechanism of radioiodine, or technetium-99m (^99m^Tc), into thyroid follicular cell or thyroid cancer cell was not very clear until the sodium iodide symporter (NIS) was finally discovered in 1996 [3]. Accumulation of radionuclide in thyroid cancer tissue or iodine avid after total thyroidectomy is dependent on the expression and activity of [10].

Function of taking up radioiodine actively can be imaged with radioiodine (such as I-123, I-131, and I-124). Gamma camera with radioiodine can visualize accurate localization of sites of pathological uptake such as metastasis lesions or residual thyroid in DTC patients who have undergone total thyroidectomy, because the lesions are highly efficient at trapping circulating iodine by expression of NIS [3].

I-131 has been successfully used for the therapy of DTC and metastatic lesions for many years. After total and subtotal thyroidectomy, radioactive iodine (RAI) as the ablation treatment is much more conducive to follow-up and monitoring of tumor recurrence. Unlike diagnostic imaging, radioiodine imaging can forecast response to therapy and can be used for theragnostic imaging, which can potentially alter the decision to treat with I-131 and finalize the subsequent therapeutic dose of I-131 [4]. RAI dose selection is generally based on patient risk factors [11]. I-131 whole body scintigraphy (^131^I-WBS) performed on days 3–10 after RAI treatment often reveals the unknown local or distant metastases. Since the release of radiation has high energy (to emit 364 keV gamma rays), ^131^I-WBS has low spatial resolution and poor image quality. In addition, a worse visualization of anatomical detail by this planar imaging makes diagnosis unclear. Hybrid SPECT/CT allows better visualization of 131I distribution within the human body and help to improve diagnostic accuracy. Chen et al. [12] reported that SPECT/CT accurately located 85.2% (69/81) and characterized 82.7% (67/81) of inconclusive lesions considered on planar imaging. 131I-SPECT/CT fusion imaging gradually becomes a popular essential examination means for the clinical staging, risk-stratifying, prognostic evaluation, and long-term follow-up of DTC [13]. It should be emphasized that routine use of radioiodine scintigraphy for surveillance is rationally used in patients with intermediate or high risk of recurrence and to assess patients for evidence of recurrence in the setting of an elevated thyroglobulin level with a negative neck ultrasonograph, but is not recommended for low-risk patients. However, it is common for 131I-SPECT/CT fusion imaging to be used in low-risk groups or only planar imaging used in high-risk groups in some domestic hospitals.

I-123, a lower 159 kev gamma emitter, has a higher counting rate compared with I-131 and provides a higher lesion-to-background signal, so I-123 scanning offers excellent image quality comparable to high-dose 131I posttreatment imaging in thyroid carcinoma patients. Moreover, with the same administered activity, I-123 delivers an absorbed radiation dose that is approximately one-fifth that of I-131 to NIS-expressing tissues [3]. I-123 scanning can decrease radiation exposure and avoid stunning and is effective for use in diagnostic radioactive iodine scans in children with DTC but is likely to miss lung lesions [14]. Using I-123 can avoid disadvantages such as stunning which is caused by previous irradiation and may reduce the therapeutic efficacy of 131I and delivery of a high radiation dose [15]. As mentioned above, I-123 not only has the same clinical value as I-131 but also has some special advantages. The clinical application of I-123 is limited by high cost due to accelerator production. Moreover, report showed that diagnostic I-123 scans undervalue the disease burden compared to I-131 scans after treatment, especially in children and in other patients with prior RAI therapy and/or distant metastasis [16]. Sarkar et al. compared the diagnostic sensitivities of 123I and 131I whole-body imaging in DTC of twelve thyroidectomized patients and found that 123I adequate for imaging residual thyroid tissue but less sensitive than 131I for imaging thyroid cancer metastases [17]. I-124 is a PET radiopharmaceutical with higher energy and a 4.2-day half-life, which potentially offers high sensitivity, better imaging characteristics, and no evidence of the stunning effect [18, 19]. In a recent study comparing the image qualities of different iodine isotopes (I-123, I-124, and I-131), I-124 showed the best imaging properties [20]. 124I PET has an advantage in the diagnosis of iodine-positive DTC or RAI avid metastatic DTC [16, 19]. Ruhlmann et al. proved a high level of agreement between pretherapeutic 124I PET and intratherapeutic 131I imaging in detecting iodine-positive thyroid cancer metastases [21]. In addition, 124I-PET proved to be a superior diagnostic tool in detecting residual, recurrent, and metastases lesions with a higher sensitivity than the conventional 131I scans [3, 22]. Hybrid imaging with 124I PET/CT is superior to 124I PET planar imaging. A study on evaluating the value of (124)I-PET/CT in staging of patients with DTC showed a lesion delectability of 56, 83, 87, and 100% for CT, (131)I-WBS, (124)I-PET, and combined (124)I-PET/CT imaging [23]. The diagnostic value of 124I PET/CT has been shown in some other studies [19]. I-124 PET/CT is superior to I-131 WBS in detecting, localizing, and differentiating between the thyroid remnant and cervical lymph node metastases and distant metastases to the lungs, liver, adrenal gland, or bone marrow [20]. However, negative 124I scan cannot help predict a negative post-131I therapy scan for patients with elevated serum Tg level and negative diagnostic 131I planar scan, and it should not be used to exclude the option of blind 131I therapy [24]. (Kist et al. performed a prospective multicenter observational cohort study to test whether (124)I PET/CT can identify patients with a tumor-negative posttherapy (131)I WBS, and they concluded that in patients with biochemical evidence of recurrent differentiated thyroid carcinoma and a tumor-negative neck US, the high false-negative rate of (124)I PET/CT after recombinant human thyroid-stimulating hormone (124)I PET/CT as implemented in this study precludes its use as a scouting procedure to prevent futile blind (131)I therapy [25].) 124IPET/CT imaging is more common than 123I scans but rarer than 131I scans, due to its cost and diagnostic reasons. Santhanam et al. [16] recommend that I-124 PET/CT imaging could be used in these clinical situations: (i) pediatric or adult patients cases with prior RAI therapy in which the I-123 scan may underestimate the disease burden; (ii) for accurate assessment of residual neck cervical disease for surgery planning; (iii) for 3D dosimetry; and (iv) detection of nonavid lesions for additional therapy (surgery, radiotherapy, and chemotherapy).

### 2.2. Other Radiopharmaceuticals in DTC and Metastases

With the development of various methodologies, such as contrast-enhanced ultrasonography (CEUS), ultrasonic-guided fine-needle biopsy, ultrasonic elastography (USE), tomography (CT), diffusion weighted imaging (DWI), and genetic mutations techniques (BRAF, RAS, RET/PTC, PAX8/PPAR*γ*, etc.), preoperative evaluation of thyroid nodules with radionuclides has rarely been used. But for DTC, many isotopes are still available for imaging patients with suspected recurrence and metastases. In addition to radioiodine, many alternative radiopharmaceuticals have been tried to identify DTC metastases, especially in cases where radioiodine fails. These radiopharmaceuticals include ^201^Tl, ^99m^Tc-sestamibi, ^99m^Tc-tetrofosmin, ^99m^Tc-depreotide, and ^111^In-octreotide which have been uniquely helpful ([26] 44). To date, the greatest attention has been paid to DTC patients that are 131I-WBS negative and Tg positive. It has been reported that Fluoro-18-deoxyglucose PET, ^99m^Tc-MIBI, ^201^Tl, and ^99m^Tc-tetrofosmin are primarily useful in the setting of a negative whole-body 131I scan and elevated serum thyroglobulin [27], particularly hybrid ^18^F-FDG PET/CT imaging.

It has been demonstrated that thyroid cancers with low iodine avidity tend to have higher glucose metabolism, which is related to reduced NIS and increased glucose transporter 1 gene expression, so 18FDG-PET/CT seems to have the highest sensitivity in this setting and may be helpful in identifying patients at higher risk or patients unlikely to benefit from additional 131I therapy. After uptake in the thyroid, 18F-FDG leads to malignancy in 30% or more of cases. Metastatic lesions without iodine avidity are a less differentiated phenotype and are prone to high glycolytic rates, which results in high glucose uptake on 18F-FDG PET [4]. Many studies have shown ^18^F-FDG PET/CT imaging can be useful for detecting recurrence or metastasizing of DTC with 131I-WBS negative and Tg positive [28–30]. Moreover, Tg level is relative to the positive rate of PET/CT scan. It is reported that ^18^F-FDG PET/CT is useful in diagnosing nonradioiodine avid DTC in patients with high levels of stimulated Tg, and the sensitivity increases with stimulated Tg levels (Tg > 28.5 ng/ml, sensitivity: 100%) [31]. Another study has reported patients with a positive PET/CT scan had significantly higher Tg values than patients with a negative PET/CT (mean 143.8 versus 26.5 ng/ml, *P* = 0.03) [32]. 18F-FDG PET/CT imaging is also effectively used for follow-up and prognosis assessment of DTC metastases and recurrence. Salvatore et al. [33] retrospectively analyzed forty-nine DTC patients with follow-up for 7.9 ± 5 years and concluded FDG-PET/CT in association with Tg normalization at short-term follow-up may be useful for long-term prognostic stratification in DTC patients. Masson-Deshayes et al. [34] showed FDG-avid lesions' number and the SULpeak owned independent prognostic value in metastatic differentiated thyroid cancer. Due to high cost and area restricted cause, 18FDG-PET/CT has been used as a complementary tool rather than a normal method in identifying the risk of death and follow-up of DTC. Herein, some other cost-effective modalities have been also referred as follows, despite their clinical practice being uncommon because of lower sensitivity and specificity compared to 18F-FDG PET/CT.

201Tl scintigraphy has been proven to be useful for detecting radioiodine-negative metastatic differentiated thyroid cancer. A study on comparing scintigraphy findings for 201Tl and FDG uptake in patients with DTC after total thyroidectomy indicates that FDG has a distribution pattern similar to that of 201Tl [35].

^99m^Tc labeled-sestamibi (^99m^Tc MIBI), or tetrofosmin, has been always used in the myocardial perfusion imaging and parathyroid imaging and is also a tumor seeking agent like pentavalent dimercaptosuccinic acid. ^99m^Tc MIBI is increasingly used to evaluate the benign and malignant of thyroid nodules. Thyroid cancer cells have been shown to take up (99m) Tc-MIBI. A recent study indicated that a ^99m^Tc-MIBI-Hot/I-123-Cold phenotype is very specific for detecting thyroid malignancy (sensitivity 52%, specificity 88%, positive predictive value 47%, and negative predictive value 90%) and suggested that patients found intraoperatively to have false-positive parathyroid scintigraphy should be evaluated for thyroid cancer [36]. Moreover, Rubello et al. suggest that a (99m) Tc-sestamibi intraoperative gamma probe can be used to identify and guide resection of recurrent locoregional tumor in DTC patients with (131)I-negative locoregional metastatic foci [37]. In addition, a study on comparing mutation analysis of cytology specimens and ^99m^Tc-MIBI thyroid scintigraphy for differentiating benign from malignant thyroid nodules in patients indicated that ^99m^Tc-MIBI scintigraphy was found to be significantly more accurate than testing for the presence of differentiated thyroid cancer-associated mutations in fine-needle aspiration cytology sample material [38]. For patients with cold thyroid nodules without ultrasound malignant suspicion and with benign/undetermined cytology, ^99m^Tc-tetrofosmin scan may be useful in the therapeutic decision of surgery [39]. However, both tracers are of limited use for the detection and monitoring of neoplastic lesions because they often generate false-positive results [40].

DTC express somatostatin receptors (SSTRs), so, (111)In or (99m) Tc-labeled somatostatin receptor analogues, can be used for DTC patients detection. Depreotide is a ^99m^Tc-labeled somatostatin analog, which binds with high affinity to SSTRs 2, 3, and 5 [41]. A study of ten radioiodine-negative patients with suspicion of recurrent or metastatic thyroid cancer were investigated with (99m) Tc-depreotide scintigraphy and (18)F-FDG-PET. Ultrasonography and/or computed tomography confirmed meanwhile selected cases, together with cytology or histological examination. The results indicated being true-positive in nine patients (90%, 9/10) with (99m) Tc-depreotide scintigraphy and in seven patients (70%, 7/10) with (18)F-FDG-PET. The former gave high specificity in terms of detection of recurrent or metastatic disease compared with (18)F-FDG-PET [42]. In addition, Stokkel et al. have focused on the use of Indium-111-octreotide scintigraphy (SRS) in DTC patients with increased Tg levels and negative I-131WBS and showed this somatostatin analogue labeled with Indium-111 revealed a sensitivity of 82% for the detection of distant metastases [6]. Another report showed that the overall sensitivity of (111)In-octreotide scintigraphy for the detection of nonfunctioning DTC metastases was 74% and the uptake seems to correlate with prognosis and survival [43]. The value of SSTR imaging in the diagnosis of DTC metastasis or recurrence is highly appreciated. However, few opposites show that somatostatin receptor scintigraphy has a limited role in imaging for recurrent or metastatic differentiated thyroid carcinoma [27].

### 2.3. Radionuclide Imaging in DTC Dedifferentiation Lesions and Refractory Lesions

Some DTC are dedifferentiated into poorly differentiated thyroid cancers (PDTC) during treatment. Dedifferentiation is related to upregulation of GLUT1 and increased proliferation [44]. A small fraction of DTC and almost all PDTC have more aggressive tumor biology with reduced or loss of NIS expression/function, hence rendering radioiodine-based diagnosis and treatment ineffective [45, 46]. It has been reported that approximately one-third of all DTC do not concentrate radioiodine and have poor prognoses [47]. Another literature showed that 20–40% of the patients with recurrent thyroid cancer or nodal metastases lose their ability to accumulate radioactive iodine due to tumor cell dedifferentiation [48]. In addition, two-thirds of patients with distant metastases ultimately develop radioiodine refractory disease [4]. For these patients, iodine whole body scan (^131^I-WBS) is negative and they cannot benefit from iodine treatment. In this case, alternative imaging modalities are needed, such as 18F-FDG PET/CT, MRI, and 18F-FDG PET/MRI [48].

18F-FDG PET/CT is used most frequently in the surveillance of iodine-refractory lesions with increased thyroglobulin level after therapy [49]. A study showed that in non-radioiodine-avid/radioiodine therapy refractory thyroid cancer patients, peptide receptor radionuclide therapy (PRRT: (90)Yttrium and/or (177)Lutetium labeled somatostatin analogs) is a promising therapeutic option with minimal toxicity, good response rate, and excellent survival benefits, and (68)Ga somatostatin receptor PET/CT is used to determine the somatostatin receptor density in the residual tumor/metastatic lesions and to assess the treatment response [50]. The RAIR (131IWBS–negative/thyroglobulin-positive) metastatic lesions can be traced using ^99m^Tc-3PRGD2 imaging, meaning these lesions are highly neovascularized. ^99m^Tc-3PRGD2 angiogenesis imaging can be used for the localization and growth evaluation of RAIR lesions, providing a new therapeutic target and a novel imaging modality to monitor the efficacy of certain antiangiogenic therapy [51].

Hürthle cell carcinoma (HCC) is a rare DTC with a tendency to develop in soft tissues of the neck and other distant sites metastases with a lower survival rate [52]. Bomanji et al. [53] have reported a combination of 131I and 99Tcm-tetrofosmin imaging may be useful to assess the extent of disease in patients with recurrent Hürthle cell carcinoma. Recently, Ga-68-PSMA PET/MRI showed abnormal PSMA uptake in the thyroid gland prompted USG-guided FNAC which revealed Hurthle cell neoplasm [54].

## 3. Nuclear Molecular Imaging in Medullary

Thyroid cancers (MTC) are rare neuroendocrine tumor derived from the thyroid C cells and produce calcitonin and carcinoembryonic antigen (CEA). It may be both as a sporadic form (75%) and as a hereditary form (25%) as part of multiple endocrine neoplasia (MEN) type 2(MENIIA and MENIIB), due to germline mutations in the RET protooncogene [55–57]. It is generally recognized that MTC can be cured only by complete resection of the thyroid tumor and any locoregional metastases [55]. Patients with MTC have high survival rate (5 years: 92%; 10 years: 87%) [58]. MTC is an indolent disease with patients frequently presenting with metastatic disease preceding the onset of symptoms [46]. Moreover, MTC is not responsive to neither radioactive iodine therapy nor thyroid stimulating hormone suppression.

Most MTC cells did not concentrate 131I, while many other molecular markers labeled by radionuclide are used in a wide range of applications like diagnosis, treatment, and follow-up of MTC patients. Although many other inspection methods replaced radionuclide imaging in early detection, in the preoperative staging, nuclear molecular imaging is essential both for preoperative staging of MENII and for postoperative restaging to detect persistence. When there are elevated levels of serum calcitonin and the conventional imaging has negative results, nuclear molecular imaging techniques including 201Tl, ^99m^Tc-(V)-DMSA, ^99m^Tc-sestamibi, ^99m^Tc-tetrofosmin, 123/131I-MIBG, Indium-111-octreotide, and ^99m^Tc-EDDA/HYNIC-TOC scintigraphy can be used [46, 59]. Compared to the above methods using a gamma scintillation camera for patients with neuroendocrine tumor imaging, the new positron emission tomography (PET/CT) methods (18F-FDG-PET, 18F-DOPA, 18F-fluorodopamine, 68Ga-DOTATOC/-NOC/-TATE, 11C-5-hydroxytryptophan) are much more chosen, due to higher sensitivity and more accuracy [60].

Although 18F-FDG is not the tracer of good choice to study well differentiated neuroendocrine tumors, it has shown a higher sensitivity in patients with MTC when compared to single photon emission tracers [57]. 18F-FDG-PET/CT is used for restaging of MTC to detect tumor recurrence. Its overall sensitivity ranges from 47% to 79% and its lesion-based sensitivity is even higher between 76% and 96% [46]. In metastatic MTC patients, 18F-FDG-PET/CT provides a useful contribution mainly in evaluating lymph node involvement whereas (111)In-Octreotide SPECT can contribute to the detection and somatostatin receptor characterization especially of bone lesions [61]. Another study also indicated that 18F-FDG PET is more sensitive than CT, MRI, and 131I-MIBG in localizing lymph node involvement in MTC patients with postsurgically elevated calcitonin levels [62].

18F-DOPA PET/CT enables early diagnosis of MTC patients with distant metastasis. In a study done by Archier et al., (18)F-DOPA PET/CT was positive in 65 of the 86 patients (patient-based sensitivity: 75.6%), and distant metastatic disease (M1) was seen in 29 patients, including 11 with previously unknown metastases revealed only by PET/CT. But F-DOPA PET/CT has a limited sensitivity in the detection of residual disease (lesion-based sensitivity: 24%) [63]. Literature shows that MTC diagnostics contemporary method (18F-DOPA) is more sensitive than conventional ^99m^Tc-(V)-DMSA method and is similar to 18F-FDG or computed tomography and magnetic resonance [60]. But some studies suggest that the sensitivities of both 18F-DOPA PET/CT and 18F-FDG-PET/CT to detect MTC are associated with calcitonin and CEA doubling time. Short calcitonin and CEA doubling times are considered the best available indicators to assess recurrence and mortality [64]. Koopmans et al. used both lesion-based and patient-based analysis to confirm that MTC lesions are best detectable when serum calcitonin is >500 ng/L and 18F-DOPA PET is superior to 18F-FDG PET, DMSA-V, and morphologic imaging, whereas with short calcitonin doubling times (< or = 12 months), 18F-FDG PET may be superior [65]. In addition, another study showed that doubling times were less than 24 months in 77% (*n* = 5 10/13) of 18F-FDG PET-positive patients, whereas 88% (*n* = 5 22/25) of 18F-FDG PET-negative patients had doubling times greater than 24 months (*P* < 0.001). Between doubling times and 18F-DOPA PET positivity, no significant correlation existed, but 18F-DOPA PET detected significantly more lesions (75%, 56/75) than did 18F-FDGPET (47%, 35/75) in the 21 patients included in WBMTB analysis. Thus, 18F-DOPA PET is much more important to assess residual disease whereas 18F-FDGPET can more accurately identify patients with progressive disease [64]. 18F-DOPA PET/CT accurately detects metastases in MTC patients with occult disease whereas 18F-FDG PET/CT may be more feasible in patients with an unstable CEA doubling time. In patients with an unstable calcitonin level, both methods are complementary [66].

MTC as a neuroendocrine tumor may produce different peptides and express their receptors, such as somatostatin receptors, gastrin/cholecystokinin-2 (CCK-2), glucagon-like peptide 1 (GLP-1), or calcium-sensing receptors. 68Ga labeled somatostatin analogues (68Ga-DOTA-TOC or 68Ga-DOTA-NOC) are a promising tool for evaluation of the expression of somatostatin receptors in patients with metastatic neuroendocrine tumors who are planned to go through therapy with 177Lu- or 90Y-labelled DOTA-TATE [57]. A study has shown that 68Ga-DOTATATE PET/CT had a sensitivity of 72.2% (13 of 18 patiens) in detection of MTC recurrence, slightly lower than 18F-FDG imaging. Despite this, 68Ga-DOTATATE PET/CT can be a useful complementary imaging tool and may identify patients suitable for consideration of targeted radionuclide somatostatin analogue therapy [67].

New approaches using gastrin receptor scintigraphy are promising because of the high expression of the CCK-2 receptor in MTCs [65]. Several ligands for the CCK2 receptor (CCK2R) have been developed for radionuclide targeting of MTC and small cell lung cancers [68]. The presence of CCK-2 receptors was used for localizing MTC and its metastases. Gastrin receptor scintigraphy seems to have higher specificity and positive predictive value but lower sensitivity than SRS [69]. Barbet et al. [70] used a pretargeting method, based on bispecific antibodies and low molecular weight radiolabeled bivalent haptens on patients with elevated circulating calcitonin after resection of primary MTC, and immunoscintigraphic was then performed 2, 5, and 24 hr after hapten injection. The result showed that pretarget immunoscintigraphic detected high-activity uptake sites in 21 of 29 (72%) patients with occult disease, including small tumor lesions in the liver. The author concluded that the use of immunoscintigraphic and guided surgery would improve the therapeutic management of recurrent MTC. Pretargeting techniques have been developed to improve radioimmunotargeting of tumors. The combination of the specificity of antibody targeting and the sensitivity of PET is very promising [71]. Earlier clinical studies reported a high sensitivity of pretargeted immunoscintigraphic using murine or chimeric anticarcinoembryonic antigen (CEA), bispecific antibody (BsMAb), and peptides labeled with 111In or 131I in MTC. Recently, a study reported that optimized pretargeted (parameters: BsMAb/peptide mole ratio of 20 and 30 h pretargeting delay) anti-CEA immuno-PET in relapsed MTC patients obtains high tumor uptake and contrast [72].

## 4. Nuclear Molecular Imaging in ATC

Anaplastic carcinoma, one of the most aggressive solid tumors, accounts for 10% of thyroid cancers or less and is characterized by a rapid growth rate and painful enlargement [1]. All anaplastic thyroid cancers do not concentrate radioiodine and have poor prognoses [47]. A mortality rate is up to 100% and median survival is less than 5 months [73]. ATC may arise de novo, but in most cases it develops from a preexisting WDTC, especially the follicular subtype [74]. Recent studies based on next generation sequencing techniques have provided further evidence to support a stepwise tumor progression from well-differentiated to poorly differentiated and eventually to ATC [75]. ATC may represent a terminal dedifferentiation of DTC. To date, there is no effective treatment for it. As well as refractory thyroid cancer, PET imaging is still the main current method. A study showed 18F-FDG-avid cases were found the lowest in DTC, intermediate in PDTC, and the highest in ATC [44]. So 18F-FDG PET/CT is presently the important method of visualization for ATC, because of its diagnostic efficiency.

Nowadays, RNA interference techniques are promising in gene therapeutic approaches. Li et al. [76] developed a triblock dendritic nanocarrier, polyamidoamine-polyethylene glycol-cyclicRGD (PAMAM-PEG-cRGD), as an siRNA vector targeting the human ether-à-go-go-related gene (hERG) in human anaplastic thyroid carcinoma cells. The study indicated that siRNA was successfully transferred to the target cells and knocked down hERG that inhibited cell growth and induced apoptosis in ATC cells in vitro.

In fact, the key role of RNAi therapy for thyroid carcinomas stimulated the search for methods that could enhance NIS expression and migration to the plasma membrane in tumor cells. Drugs such as retinoic acid and, discovered more recently, mTOR, BRAF, and MEK inhibitors can inhibit the intracellular kinases responsible for both tumor progression and NIS disappearance. The patient is benefited by both tumor stabilization and RAI treatment, with the internal radiation killing tumor cells resistant to the kinase inhibitors [77]. Otherwise, with transfer of the NIS gene into cells without NIS gene expression (such as therapeutic cells: cytotoxic T or natural killer cells; or Dedifferentiated cancer cells, etc.), the NIS-expressing cells can be imaged by radionuclide-based molecular imaging techniques using gamma ray or positron-emitting radiotracers and be cleared by beta or alpha particle-emitting radionuclides [3]. Recently, a study on the expression and function of NIS modulated by miRs demonstrated that miR-339-5p may play a role in decreasing hNIS-mediated RAIU in follicular thyroid tumors but not in papillary thyroid tumors, and since miR-195 is not upregulated in papillary thyroid tumors, it does not directly contribute to the reduction of levels of NIS in papillary thyroid tumors [45]. This also confirms the potential for high-risk follicular cancer to develop into ATC.

## 5. Novel Nuclear Molecular Imaging in Thyroid Carcinoma

### 5.1. Radio-Immunoimaging in Thyroid Cancer

Radio-immunoimaging is a method of labeling radioisotopes to highly specific Mab by special methods and imaging with high sensitivity and high resolution SPECT/CT or PET/CT. Immunoimaging is a noninvasive, quantitative scan for getting sophisticated information to target molecules, unlike immunohistochemistry in single biopsy, which can provide associated information of Mab targeting against target site and dosimetric determinations before radioimmunotherapy (RIT) [78]. Many isotopes, such as ^99m^Tc, ^124^I, ^64^Cu, ^89^Zr, and ^68^Ga, have been used for thyroid nodule or cancer radio-immunoimaging [72, 79–83]. Conventional thyroid scintigraphy does not allow the distinction among benign and malignant thyroid proliferations. It is reported that well-differentiated thyroid carcinomas almost invariably express galectin-3 and galectin-7, while benign thyroid proliferations do not, so expression of galectin-3 and galectin-7 in thyroid malignancy may be as potential diagnostic indicators. In fact, other group confirmed that, in addition to galectin-3, there is no significant adjunct diagnostic value in Gal-7 for thyroid malignancy [84]. Bartolazzi et al. used galectin-3 based thyroid immunoscintigraphy in 38 mice with tumor mass and found that the group of human galectin-3 positive thyroid cancer xenografts (ARO) showed an optimal visualization between 6 and 9 hours from injection of the radiotracers, while Galectin-3 negative tumors were not detected at all [83]. Wagner et al. [82] studied radiolabeled antibody [(64)Cu]Cu-NOTA-D13C6 on mice for immuno-PET imaging and reported that it represents a novel and promising radiotracer for radioimmunoimaging of PDGFRalpha in metastatic papillary thyroid cancer. There are few radioimmunoimaging agents that entered the clinical trial. It is reported that (89)Zr-cmAb U36 has been used in patients with head and neck squamous cell carcinoma (HNSCC), including thyroid cancer, to quantitatively assess biodistribution, uptake, organ residence times, and radiation dose [79]. But to date, the majority of immuno-PET imaging are still at the experimental stage, due to human anti-rat antibody (HAMA) effect, poor image contrast, and inevitable false negative problem.

### 5.2. Nanomaterials Mediated Nuclear Imaging in Thyroid Cancer

Many new nanomaterials have emerged as a particularly fascinating area of widespread interest in molecular imaging, drug delivery, and therapy. Some well-studied nanomaterials include quantum dots (QDs), dendrimers, nanotubes, micelles, gold nanoparticles, and nano/microbubbles [85]. One animal study showed that (99m)Tc-Sb(2)S(3) with 50 nm particles, in the dosage of 0.01 ml or 0.02 ml, could be good choice for Sentinel Lymph Node Biopsy (SLNB) of thyroid cancer [86]. In recent years, some nanomaterials have been used clinically. The application of nanocarbon in the sentinel lymph node of thyroid cancer is a model. Carbon nanoparticles have proved to identify parathyroid tissue and protect it in thyroid cancer surgery [87, 88]. In addition, utilizing the PDA shell, radionuclide 131I could be easily labeled onto single-walled carbon nanotubes (SWNT@PDA-PEG) and used for nuclear imaging and radioisotope thyroid cancer therapy [89].

Nanotransporter is an ideal gene transfer vector for gene therapy. Potential dual purposes of imaging and targeted drug delivery of nanoparticles (NPs) brought great prospect to targeted treatment. However, NPs are not permeable to cytoplasmic membrane, so exploring methods for NPs uptake into cells is critically important. Surface modifications of NPs have emerged, such as polyethylene glycol complexing or labeling with CPP [85]. To improve the efficiency of delivery, some tissue-specific antibodies have been conjugated to NPs. Watanabe et al. [90] reported that conjunction of QDs reacted with 1-ethyl-3-(3-dimethylaminopropyl) carbodiimide hydrochloride and N-hydroxysulfo-succinimide in 2-(morpholino) ethanesulfonic acids and JT95 IgM antibodies could specifically detect the thyroid carcinoma associated antigen, with 91.4% sensitivity and 90.0% specificity. Another study showed that a nanodelivery system (TSH-nanoliposomes) could increase the intracellular uptake of NPs in cells expressing the TSHr. Furthermore, TSH-nanoliposomes encapsulated with gemcitabine showed improved anticancer efficacy in vitro and in a tumor model of follicular thyroid carcinoma [91]. CPPs are a powerful tool for transporting diverse materials across the cell membrane [92]. Josephson et al. first reported that cellular uptake of iron oxide nanoparticles covalently conjugated with CPP (Tat) is increased [85]. CPP could mediate nanoparticle delivery in stem cells [85]. Recent views show that cancer stem cells can be considered as a potential therapeutic target in thyroid carcinoma [93]. Thus, CPP mediated nanocarrier may provide a new way for the treatment of thyroid cancer [94]. Unfortunately, the vast majority of studies in this area are in the experimental stage.

### 5.3. Optical Imaging in Thyroid Cancer

Recently, I-131 and I-124 were reported to have sufficient energy to result in Cerenkov radiation that can be visualized with sensitive optical imaging equipment, and cells transfected with NIS gene were successfully imaged with the radioiodine using an optical imaging instrument in an in vivo animal model. This Cerenkov luminescence imaging (CLI) can provide a new optical imaging (OI) strategy in preclinical thyroid studies [95].

## 6. Conclusion

In summary, different histological types of thyroid cancers have great difference in biological behaviors and prognoses. Nuclear molecular imaging plays an important role in the evaluation and management of different types of thyroid cancer, especially in detecting residual, recurrences, and metastases, helping patients to get the optimal medical therapy for their diseases. But so far, there is no single sensitive diagnostic imaging method to reveal all lesions, so it is conducive to optimize the imaging method by recognizing the biological characteristics and pathological types of thyroid cancer. Meanwhile, complementing different imaging methods can also improve sensitivity and specificity. With the development of nuclear medicine molecular imaging, more potential imaging methods will emerge, ultimately achieving accurate diagnosis and personalized treatment.
